# Early experience with low-pass filtered images facilitates visual category learning in a neural network model

**DOI:** 10.1371/journal.pone.0280145

**Published:** 2023-01-06

**Authors:** Omisa Jinsi, Margaret M. Henderson, Michael J. Tarr

**Affiliations:** 1 Department of Psychology, Carnegie Mellon University, Pittsburgh, Pennsylvania, United States of America; 2 Neuroscience Institute, Carnegie Mellon University, Pittsburgh, Pennsylvania, United States of America; 3 Department of Machine Learning, Carnegie Mellon University, Pittsburgh, Pennsylvania, United States of America; Hanyang University, REPUBLIC OF KOREA

## Abstract

Humans are born with very low contrast sensitivity, meaning that inputs to the infant visual system are both blurry and low contrast. Is this solely a byproduct of maturational processes or is there a *functional* advantage for beginning life with poor visual acuity? We addressed the impact of poor vision during early learning by exploring whether reduced visual acuity facilitated the acquisition of basic-level categories in a convolutional neural network model (CNN), as well as whether any such benefit transferred to subordinate-level category learning. Using the ecoset dataset to simulate basic-level category learning, we manipulated model training curricula along three dimensions: presence of blurred inputs early in training, rate of blur reduction over time, and grayscale versus color inputs. First, a training regime where blur was initially high and was gradually reduced over time—as in human development—improved basic-level categorization performance in a CNN relative to a regime in which non-blurred inputs were used throughout training. Second, when basic-level models were fine-tuned on a task including both basic-level and subordinate-level categories (using the ImageNet dataset), models initially trained with blurred inputs showed a greater performance benefit as compared to models trained exclusively on non-blurred inputs, suggesting that the benefit of blurring generalized from basic-level to subordinate-level categorization. Third, analogous to the low sensitivity to color that infants experience during the first 4–6 months of development, these advantages were observed only when grayscale images were used as inputs. We conclude that poor visual acuity in human newborns may confer functional advantages, including, as demonstrated here, more rapid and accurate acquisition of visual object categories at multiple levels.

## Introduction

The trajectory of maturation in the visual system mirrors the trajectory of infant development more generally, with adult-like abilities emerging only over months or years [1]. Given that neonates of many other mammalian species begin life with relatively high-quality vision, it is curious that human infants start life with such poor vision. Under one view, the human altricial state at birth is attributed to our extreme intelligence (and by consequence, large brain), which creates selective pressure for humans to be born in a relatively premature, under-developed state [2]. However, an alternative view holds that the premature starting point and path of human development is, at least in part, a functional adaptation that helps bootstrap many mental abilities [3].

**Fig 1:**
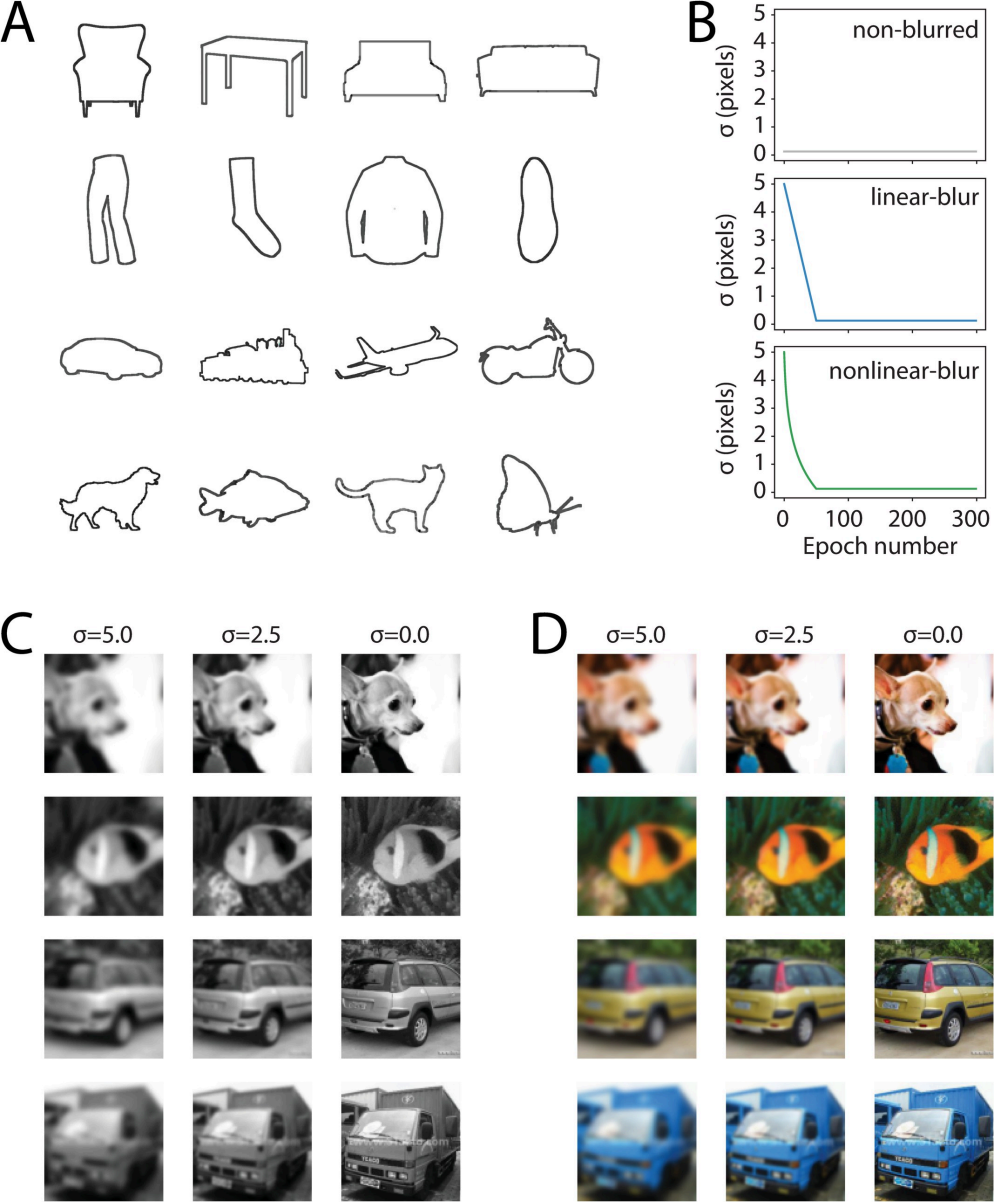
Rationale and experimental design. **(A)** Rosch et al.’s stimulus images depicting the global shape of exemplars from four visually-similar basic-level categories within four superordinate categories (rows; adapted from Fig 1, [4]). **(B)** Schematics illustrating how blurring (in terms of the parameter sigma or σ; in units of pixels) was manipulated across three conditions in Experiment 1. In the non-blurred condition (top), sigma was fixed at a small value that results in no blurring throughout training, while in the two blur conditions, sigma started at 5 pixels and decreased—at different rates—over the first 50 epochs of training, corresponding to an increase in spatial acuity over the course of learning. See *Methods* for details. **(C-D)** Example ecoset images from the basic level categories “dog”, “fish”, “car”, “truck”, blurred at three example values for sigma. Note that larger sigma values give rise to blurrier images, while σ = 0 results in an intact, non-blurred image. Examples of the same images are shown for the **(C)** grayscale and **(D)** color conditions.

Consistent with the latter account, our hypothesis is that poor visual acuity at the earliest stages of development facilitates the infant’s acquisition of basic-level object categories. As an early learning objective, basic-level categories are at the core of the acquisition of stable mental concepts and foundational for naturally organizing large numbers of similar objects into behaviorally-relevant semantic clusters (e.g., “apple”, “table”, “bird”, “fish”, etc.; [4]). In their seminal paper, Rosch et al. [4] propose that basic-level categories are those that maximize within-category similarity, while minimizing across-category similarity. Moving up or down the hierarchy, superordinate categories (e.g., “animal”) will have a lower degree of within-category similarity, while subordinate categories (e.g., “red-tailed hawk”) will have a higher degree of across-category similarity. Rosch et al. further suggest that object shape, defined as the overall outline or silhouette of objects once their orientations have been aligned and their size normalized (Fig 1A), should exhibit a correlational structure that reflects the organization of objects into basic-level categories. This relationship predicts improved ability to infer basic-level category representations if images are transformed in such a way as to emphasize global shape and reduce non-shape and fine-grained shape information.

To examine this hypothesis, we simulated the process of basic-level category acquisition using neural network models trained on large-scale image datasets. We exploited the fact that global shape information can be enhanced by low-pass filtering images (blurring), which removes fine-scale spatial detail, but retains coarse-scale shape features. Below we provide additional background on the relationship between image blurring, silhouette information, and basic-level categories, as well as a review of relevant work that has examined the effect of image blurring on category learning in somewhat different domains.

### The role of global shape in basic-level categorization

Multiple studies lend support to the proposal that information about basic-level categories is carried by the coarse outlines/silhouettes of objects—which roughly correspond to the outputs of low-pass filtered versions of images. Cutzu and Tarr [5] used a simple computational model of silhouette similarity and found that object views within a basic-level category are more similar to one another than are views of objects between categories (see also [6]). Cutzu and Tarr suggest that human infants may perform binary basic-level categorization tasks (e.g., cats vs. dogs) based primarily on the information carried by object silhouettes. This prediction was borne out by Quinn, Eimas and Tarr [7], who found that 3 and 4-month-old infants were able to form, in a pairwise discrimination task, categorical visual representations for cats versus dogs based only on object silhouettes. This finding lends credence to the hypothesis that global outline shape may play a critical role in basic-level categorization. Reinforcing this point, French et al. [8] observed that using input images filtered to remove high spatial frequencies—thereby emphasizing outline shape over finer image details—improved an autoencoder’s ability to discriminate between the same cat and dog images as used in [7].

One limitation of both of these studies is that only a pairwise discrimination between two categories was tested. Consequently, it is possible that the visual features preserved in the low spatial frequencies of these images are able to support the discrimination of these particular 18 cats and 18 dogs, but do not generalize to the more complex space of discriminating between multiple basic-level categories. A second limitation is that both studies used images of single objects against white backgrounds. As a result, the task did not require visual object segmentation or figure-ground processing and, consequently, the stimulus images were biased towards outline shape beyond what would be expected from natural images (which depict objects in the context of complex visual environments; e.g., Fig 1C and 1D). Thus, while these two studies are consistent with our hypothesis, they leave open many questions regarding whether the information carried by global object shape is privileged with respect to acquiring basic-level category knowledge. We address these limitations using a large-scale simulation of visual category learning that requires discriminating between over 500 object classes.

### The perception of visual object shape

Stepping back, it is axiomatically true that the human visual system does not process images to extract the global shapes or silhouettes of objects. The visual system instead includes a complex hierarchy of overlapping spatially tuned neurons, the earliest of which respond to roughly circular regions of space and serve as *spatial frequency* filters [9] (or as construed by Marr & Hildreth [10], a set of multiscale edge detectors which measure local intensity changes which are then combined into a global description of the image). Thus, as an approximation to global shape, a population of appropriately tuned neurons will produce a low-pass filtered image which lacks fine-grained details and which will highlight local regions of high contrast. At later stages of the object recognition hierarchy, progressively more complex features emerge across anterior regions of visual cortex, including sensitivity to class-diagnostic object features or specific categorical domains (e.g., faces, places, bodies, food, tools, etc.). Interestingly, the same feature hierarchy appears to be reflected in modern neural networks performing object classification: multiple studies have established that early layers of such networks best account for variance in early visual areas while later layers of the same networks best account for variance in high-level and domain-specific visual areas [11, 12].

In this context, we propose that basic-level categorization within the infant visual system is facilitated by a bias in the representation of spatial frequency. That is, encoding low-frequency information at the expense of high-frequency information serves to emphasize global shape and high contrast shape boundaries—a bias in infant vision that can be functionally approximated by low-pass filtering of input images. We view low-pass filtered images as better ecologically-anchored relative to the simplified silhouette-like stimuli that have been used in past work in that silhouettes omit all interior surface and shape information and do not reflect the likely outputs of early visual processing in infants.

### Changes in visual abilities across development

Compared to adults, human newborns are much less sensitive to high spatial frequencies but grow progressively more sensitive to high spatial frequencies (and more adult-like) over the course of development [13, 14]. Spatial acuity improves substantially over the first year of life, with studies reporting near-adult acuity levels in infants between 6–12 months of age (although acuity continues to improve until around 3 years of age; [1, 15]). More generally, as reviewed by Brown and Lindsey [13], sensitivity to light, color, and contrast are all much lower in infants than as measured in adults. Underlying these limitations, infant contrast sensitivity is incredibly poor, measuring 50 times lower than adults at three months of age. Relevant to the manipulations used in our study, there is also evidence that the infant contrast sensitivity function is not only lower, but is shifted to lower spatial frequencies. Similarly, color vision in the first few months of life is poor relative to adult abilities [13, 15, 16]. At the same time, chromatic sensitivity appears to mature earlier than acuity, with 4–6 month-old infants showing adult-like hue discrimination and categorization performance [17–19] and 6–8 month-old infants demonstrating use of canonical colors in object recognition [20]. Irrespective of the rate of maturation across different visual abilities, at birth human infants experience a relatively blurry and colorless world that only improves over the course of development.

### Reducing input complexity

From a theoretical perspective, the initial prematurity of human infants as well as their overall path of development may facilitate more effective learning across a variety of domains [3]. In vision, we hypothesize that the trajectory of visual development discussed above serves as a functional constraint that provides an advantage for learning foundational knowledge about the world in the form of visual categories. This proposal is an instance of the *Starting Small* principle put forward by Elman [21]: that the reduction in input complexity in infant vision—as a consequence of the path of visual development—enables both faster and better category learning. Intuitively, our argument is as follows. Basic-level categories form the conceptual scaffolding for much of our semantic knowledge [4]. Consequently, we assume that a core objective of early human development is to acquire robust basic-level categories—leaning heavily on visual experience. Yet contrary to this goal, the visual world presents as a complex, highly detailed environment. While some attributes of this environment help to specify the basic-level structure of the world—through shared features across within-category exemplars—other attributes *detract* from learning this structure. In particular, fine-grained details of objects such as metric shape and, especially for non-living things, colors or surface textures, often vary for exemplars within a category. As such, fine-grained features frequently *increase* the dissimilarity between items within basic-level categories. Thus, object information carried by high spatial frequencies and/or color may be detrimental to learning robust categories.

How then, does the infant learner select visual attributes appropriate to the learning objective? One possibility is that selective attention serves to orient the infant to the most informative visual attributes in inputs. While such biasing might be theoretically achievable, it would require a great deal of neural machinery—a complex system devoted to identifying, orienting, and selecting across complex visual inputs. In contrast (sic), somewhat the same end goal may be achieved by reducing input complexity via limited contrast sensitivity at birth (but allowing contrast sensitivity to improve over the course of development as fine-grained information becomes relevant to more subtle, adult-like visual tasks). Under this view, a reduction in complexity is a consequence of the way in which visual percepts are processed by the infant’s maturing visual system. Supporting this point, Brown and Lindsey [13] present evidence that infant contrast sensitivity is limited as a consequence of mid-level visual processing constraints and *not* as a consequence of any attentional effect in alert infants. As such, human infants are fully capable of visually exploring their surrounding environment (and, as a result, acquiring category knowledge), but their inputs are biased towards lower spatial frequencies, high contrast, and poor color perception due to intrinsic properties of their developing visual systems.

### High-performing neural networks enable computational studies of category learning

Until recently, a variety of practical considerations made it challenging to study and manipulate visual learning in an ecologically relevant context (for recent state-of-the-art work, see [22]). While studies relying on recovered sight in older children or adults are somewhat informative, they are necessarily limited in their conclusions because of concurrent maturational changes that occur regardless of the structure of perceptual inputs [23]. However, the tools available for studying learning from experience have transformed over the last decade due to the rapid advance of artificial intelligence and computer vision in the form of deep convolutional neural networks (CNNs). CNNs are a type of feed-forward artificial neural network that are inspired by the architecture of the primate visual cortex and that have been applied to a wide variety of visual tasks, including image recognition and classification. This correspondence, along with performance that approaches human levels for many tasks [24], suggests that CNNs present a useful testbed in which to examine theories of visual learning using natural images [25]. CNNs also allow tight control over parameters of the model and the training regime (e.g., learning rate, input content, etc.). Thus, as a starting point, CNNs provide models that are high-performing approximations of human behavior for many tasks.

At the same time, it is an open question as to the degree to which learning in CNNs is built on similar computational principles to those realized in biological systems [26]. To highlight one particular concern, CNNs learn using extremely large-scale datasets—millions of distinct images—which may exceed the amount of input (at least in variety if not sheer quantity) received by human infants during their development. Note also that these datasets are often less controlled than one might like. For example, the popular ImageNet image dataset [27] contains a mixture of basic- and subordinate-level categories that does not approximate the real-world distribution of categories. Thus, training a model from scratch on solely ImageNet categories forces the network to learn both basic- and subordinate-level categories throughout training, making it both difficult to isolate the mechanisms for individual levels of category learning and to mirror the basic to subordinate progression that typifies human category learning. In contrast, ecoset, a recently released large-scale image dataset, is more ecologically motivated and only contains basic-level categories [28].

In the work we present here, we take advantage of these recent advances in models and datasets by using one of the most high-performing visual classification models, ResNet50 [29], trained on the ecoset dataset [28]. In contrast to earlier work that relied on binary discrimination tasks, we explore the impact of reducing input complexity on multi-way classification across a large number of basic-level categories using natural images (which also necessitate segmentation/figure-ground processing). As a result, the overall task we adopt is better aligned with the learning problem facing the human infant. Of note, because data collection in simulations is inexpensive and efficient as compared to human infant studies, we are able to collect data over a much wider range of input and training conditions.

### Past studies using CNNs and image blur

Related to our work, other recent studies have used CNNs to explore the effect of image blur on learning of object representations. One study by Avberšek et al. [30] used a coarse-to-fine image training regime, training CNNs initially with low-pass filtered images and then gradually introducing higher spatial frequencies as training progressed. As with other forms of learned invariance in CNNs, progressive training for a given perceptual dimension where variation is explicit (i.e., by isolating low spatial frequencies at the beginning of training) confers stronger invariance over that dimension. That is, Avberšek et al. found that models trained using a coarse-to-fine regime performed significantly better on blurred images during validation testing than models trained without blurred images. In addition, when presented with images that combined conflicting information at low- and high-spatial frequencies, models trained with the coarse-to-fine regime were found to be more sensitive to low spatial frequencies than models trained with the standard regime. However, in contrast to the results we will discuss below, these benefits only maintained if blurred images continued to be included throughout training. Furthermore, there was no representational difference between models when validated on images having full spatial frequency information.

Also related to our present study, earlier work with CNNs has focused on how blurred training images impact learning in the specific domain of face recognition. Vogelsang et al. [31] found that experience with blurred images during early training of a CNN enhanced the ability to learn invariant spatial representations, which are central to adult-like configural face processing. Similarly, Jang and Tong [32] found that a network trained with initially blurred faces and objects was invariant across blur for face recognition, but not object recognition. This dissociation appears consistent with the claim that face recognition relies on somewhat different mechanisms from generic object recognition. Interestingly, a parallel finding was recently reported by Li et al. [33], who found that low-spatial frequency preprocessing of images helps model robustness with respect to both adversarial attacks (e.g., images intentionally designed to fool a model) or image corruption (e.g., noisy images). However, neither study addressed our central question: namely whether reduced input complexity might directly affect category learning.

### Our approach

We posit that poor infant vision at birth is not altricial by accident or for purely neurodevelopmental reasons. Rather, consistent with past work, poor vision early in development may be, at least in part, a functional adaptation that bootstraps faster and more effective learning across multiple ecologically critical dimensions. We examine this hypothesis using a CNN trained to perform basic-level object categorization on the ecoset database [28]. Our use of the ecoset dataset, which is composed of a large number of objects in basic-level categories, allows us to address a critical, currently unanswered question of how basic-level category acquisition, at an ecologically relevant scale, is impacted by early experience with blurred inputs. Our findings suggest that experience with blurred images during early learning benefits the acquisition of basic-level categories—an organizing principle of conceptual knowledge that is central to adult cognition.

## Results

### Experiment 1: Background & motivation

Models were trained to perform basic-level object categorization across six conditions defined by manipulations of spatial blur and color applied to training images drawn from the ecoset dataset ([28]; Fig 1B–1D). The spatial blur manipulation assessed how the dynamics of visual acuity changes over time impact learning and recognition performance. In addition to a non-blurred image condition, we included two different time courses of blur reduction across training: blur decreasing linearly over time (linear-blur condition) and blur decreasing logarithmically over time (nonlinear-blur condition). The color/grayscale manipulation assessed whether color provides additional information with respect to object identity (i.e., some categories have highly consistent colors; [34]). This manipulation also served to compensate for the known tendency of CNNs to be biased towards color and texture rather than shape [35]. In addition, human infants have poor color sensitivity for the first 4–6 months after birth [15]. Thus, it is important to understand how color may interact with the effect of image blur.

### Experiment 1: Results

**Fig 2:**
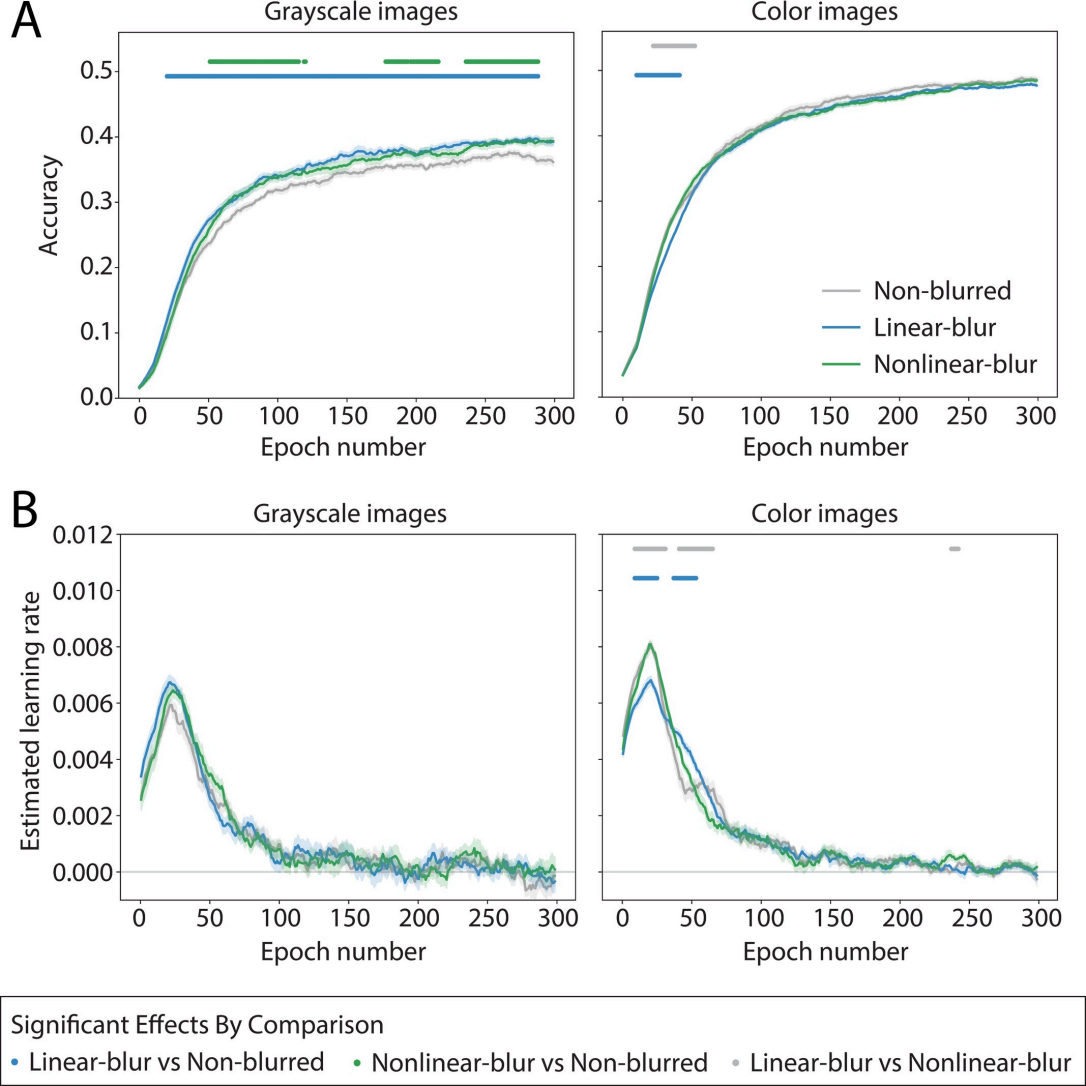
Experiment 1 model performance. Performance is shown over training for models in six conditions defined by the factors of blur and color. All models used the ResNet-50 architecture and were trained to perform basic-level object categorization using the ecoset image dataset. Each plotted point is an average across 10 runs of an otherwise identical model with different random seeds. Shaded error bars reflect mean ± SEM across these 10 trials. Dots along the top of each plot indicate time points at which a linear mixed effects model over a sliding temporal window revealed a significant effect of the specified pairwise condition comparison, in either direction (FDR corrected, ɑ = 0.05). **(A)** Validation set accuracy. Gray corresponds to models trained with *non-blurred* images, blue to models trained with images whose blur decreases linearly over the first 50 epochs (*linear-blur* condition), and green to models trained with images whose blur decreases according to a logarithmic function over the first 50 epochs (*nonlinear-blur* condition). Left and right plots show averages for models trained using either grayscale images or color images, respectively. Accuracy was temporally smoothed to reduce noise. **(B)** Estimated learning rate computed as slope of accuracy over time. Colors correspond to models as in **(A)**.

Across all spatial blur conditions, models trained on color images showed higher overall accuracy as compared to models trained on grayscale images (Fig 2; two-way ANOVA on time-averaged validation set accuracy; main effect of Color: *F*_1,9_ = 788.81, *p* = 6.72e-34, main effect of Blur: *F*_2,18_ = 2.28, *p* = 0.11, Color x Blur interaction: *F*_2,18_ = 11.34, *p* = 7.74e-5). Furthermore, the effect of blur on model performance differed between color and grayscale images. As shown in the left panel of Fig 2A, when using grayscale images, both the linear-blur and nonlinear-blur models achieved higher accuracy than models trained on non-blurred images, with linear-blur models performing slightly better than nonlinear-blur models. This difference between the linear-blur and non-blurred models manifested early in training, around epoch 25, and remained consistent throughout the remainder of training, while the difference between the nonlinear-blur and non-blurred models appeared later in training, closer to epoch 50, and was less consistent over subsequent training. No significant differences between the linear-blur and nonlinear-blur models were observed (linear mixed effects model with fixed effects of condition and epoch number, evaluated using a sliding window; significant effect of condition, FDR corrected ɑ = 0.05; for details see *Methods*). Conversely, as shown in the right panel of Fig 2A, when using color images, there was little benefit for training on blurred images, with all models converging to roughly the same level of performance by epoch 50.

Computing the learning rate of each model, estimated as the slope of accuracy over time, provides a more detailed picture of how the different conditions diverged over learning. Learning rates peaked at epoch 25, then decreased rapidly, reaching near zero by the end of training. For the grayscale models, there were no differences in learning rates across models in the different blurring conditions (Fig 2B, left). For the color models, the learning rate for the nonlinear-blur and non-blurred models each achieved a higher peak learning rate than the linear-blur models, but following this peak their learning rates decreased to a similar level as the linear-blur model (Fig 2B, right). The learning rates of all color models converged with one another by epoch 75.

### Experiment 2: Background & motivation

In Experiment 1, initial training with blurred visual inputs facilitated learning of basic-level visual categories for grayscale images. In Experiment 2, we explored whether this advantage for learning basic level categories transfers to the acquisition of categories at the subordinate level. To address this question, we fine-tuned the models from Experiment 1 to perform classification using the ImageNet dataset, which contains both basic-level and subordinate-level labeled categories.

### Experiment 2: Results

**Fig 3:**
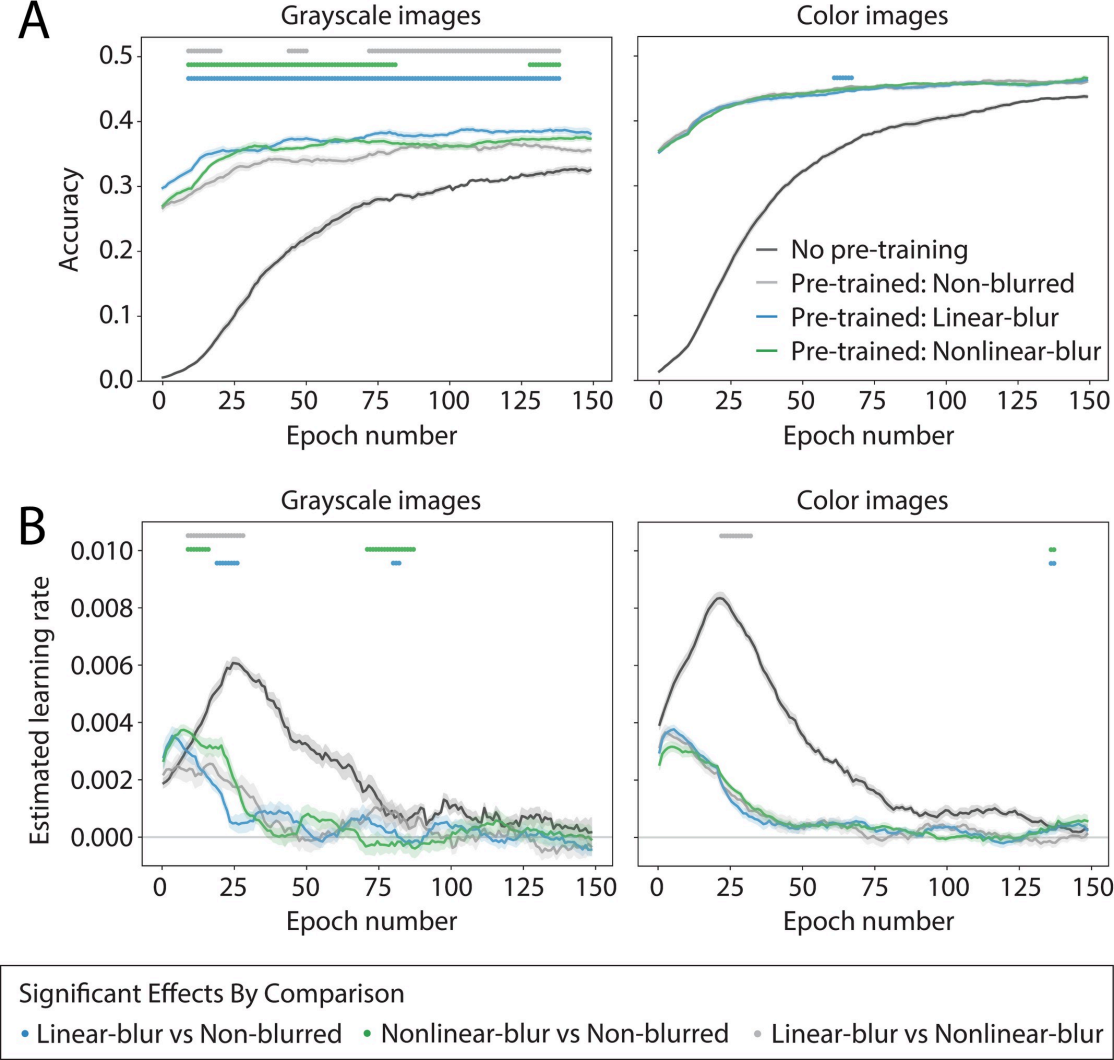
Experiment 2 model performance. Performance is shown for ecoset-trained models from Experiment 1 that were fine-tuned with ImageNet images for 1000-way object categorization. ImageNet contains labels at different category levels, with some images labeled at the basic-level and others labeled at the subordinate-level. Fine-tuning was run only with non-blurred ImageNet images. Each plotted point is an average across 10 runs of an otherwise identical model with different random seeds. Shaded error bars reflect mean ± SEM across these 10 trials. Dots along the top of each plot indicate time points at which a linear mixed effects model over a sliding temporal window revealed a significant effect of the specified pairwise condition comparison, in either direction (FDR corrected, ɑ = 0.05). **(A)** ImageNet validation set accuracy. Light gray corresponds to models trained with ecoset in the *non-blurred* condition, blue to models trained with ecoset in the *linear-blur* condition, green to models trained with ecoset in the *nonlinear-blur* condition, and dark gray corresponds to new models with no pre-training (that is, starting from scratch). Left and right plots show averages for models trained using either grayscale images or color images, respectively. Accuracy was temporally smoothed to reduce noise. **(B)** Estimated learning rate computed as slope of accuracy over time. Colors correspond to models as in **(A)**. Only pairwise comparisons between different pre-trained models are shown; all pre-trained models performed significantly better than the no-pre-training models at all time points.

As in Experiment 1, models trained with color images reached a higher overall accuracy level as compared to models pre-trained and fine-tuned with grayscale images (Fig 3, two-way ANOVA on time-averaged validation set accuracy; main effect of Color: *F*_1,9_ = 4300.13, *p* = 5.95e-66, main effect of Blur: *F*_3,27_ = 2157.61, *p* = 2.11e-70, Color x Blur interaction: *F*_3,27_ = 25.14, *p* = 3.10e-11). For both color and grayscale images, there was a general benefit of pre-training on ecoset, with all pre-trained models, irrespective of the blur condition, showing higher validation accuracy at all time points as compared to models with no pre-training. This result is not surprising given that models trained from scratch did not have the benefit of previously-learned features related to either early visual processing or basic-level categorization. Most relevant to the central question of Experiment 2 are the observed differences in performance over fine-tuning for grayscale models pre-trained with non-blurred images as compared to blurred images (Fig 3A, left). In contrast to the differences shown for grayscale models, few differences in performance over fine-tuning for color models were observed across any of the pre-training blur conditions (Fig 3A, right).

Because differences across pre-training conditions were observed only for grayscale models, we focus on these results. Across many of the time points during fine-tuning, both the linear-blur and the nonlinear-blur models reached a higher validation set accuracy as compared to the non-blurred models. This difference was more pronounced for the linear-blur models, whose performance was significantly higher than the non-blurred models at all time points (linear mixed effects model with fixed effects of condition and epoch number, evaluated using a sliding window; significant effect of condition, FDR corrected ɑ = 0.05; for details see *Methods*). In contrast, performance for the nonlinear-blur models was more variable, initially having a similar average validation accuracy as the non-blurred models, but then increasing and approaching the accuracy of the linear-blur models after 30 epochs, and then briefly decreasing to the same accuracy as the non-blurred models. The overall accuracy of the nonlinear-blur models was significantly higher than the non-blurred models for the first half of training, and again for several time points near the end of training. Finally, there were some significant differences between the linear-blur and nonlinear-blur models, with better validation accuracy for the linear-blur models.

### Experiment 2: Basic-level vs. subordinate-level accuracy

**Fig 4:**
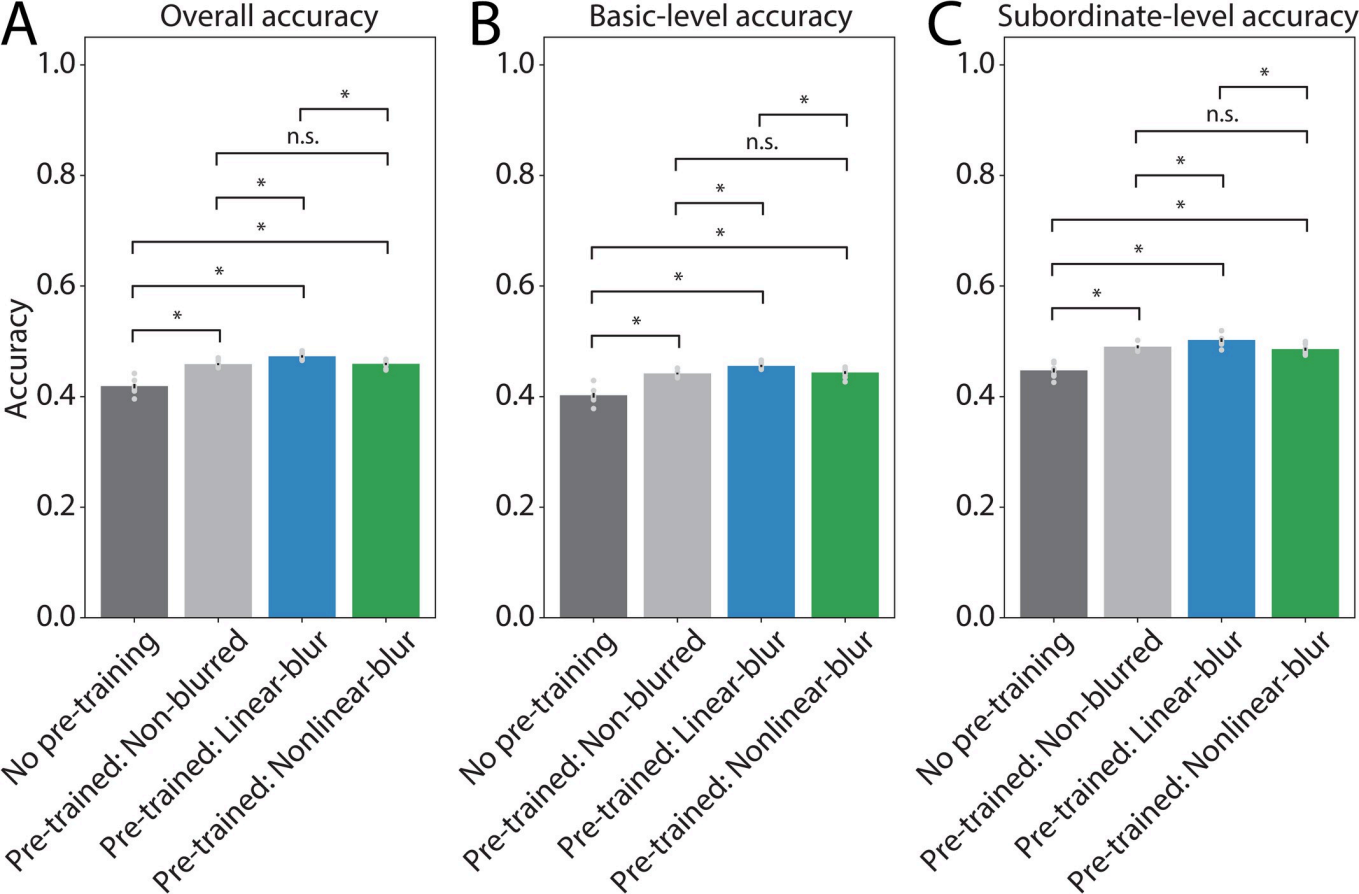
Experiment 2 model performance split by category level. Models pre-trained on grayscale ecoset images with various blur conditions were fine-tuned for 1000-way image classification on ImageNet; the performance of these models is shown separately for the basic-level and subordinate-level category labels in ImageNet. Only results from models pre-trained with grayscale images are plotted because few differences were observed between models pre-trained with color images (see Fig 3). Validation set accuracy was computed using **(A)** all categories, **(B)** basic-level labeled categories only, and **(C)** subordinate-level labeled categories only. Bar heights and error bars indicate mean ± SEM across 10 trials of each model, light gray dots show accuracy for individual trials. Brackets above bars indicate the significance of pairwise comparisons between conditions, assessed using a two-tailed independent samples *t*-test for each pairwise condition comparison (FDR corrected, ɑ = 0.05) where * denotes a significant difference in either direction and n.s. denotes no significant difference.

The results of Experiment 2 establish that pre-training with blurred ecoset images in a basic-level categorization task facilitates transfer to classifying ImageNet images in what is, effectively, a combined basic-level and subordinate-level classification task. Importantly, the magnitude of the benefit of pre-training was larger for pre-training on blurred versus non-blurred ecoset images. Given that ImageNet is composed of both basic- and subordinate-level category labels, this benefit may be driven solely by the presence of basic-level categories within ImageNet. Alternatively, representations learned during the acquisition of basic-level categories with blurred inputs may additionally support subordinate-level categorization. To address these alternatives, we hand-labeled the 1000 categories in ImageNet as either basic or subordinate and then re-computed the validation set accuracy for each of our grayscale models split by basic and subordinate labeled images. Fig 4 shows these results for the basic/subordinate category level split (panels B-C) as well as across all categories (panel A). This analysis replicated the results of the previous analysis (Fig 3), in that the three pre-trained conditions each showed significantly higher accuracy than the from-scratch models, and of the pre-trained conditions, the linear-blur models showed significantly higher accuracy than either the non-blurred models or the nonlinear-blur models. Importantly, these differences in accuracy were found for both basic- and subordinate-level categories alone, as well as for their aggregate. Thus, our current results indicate that the benefits of early experience with blurry images generalize from basic-level categorization to subordinate-level categorization.

## Discussion

Human visual cognition is grounded in our knowledge of basic-level object categories [4] and the rapid and stable acquisition of these categories in early development is central to building robust world knowledge. It is our hypothesis that the ability to learn basic-level categories may be improved by reducing the complexity of visual inputs during early learning. We examined this hypothesis by training a neural network model with a regime in which inputs were initially low-pass filtered and became progressively less blurry over time. Because blurring images emphasizes global object shape information and reduces fine-grained shape and texture information, we predicted that early experience with blurred images would improve category learning. This prediction was confirmed across both of our experiments. In Experiment 1, training on blurred images improved the accuracy of learning for basic-level categories relative to training with non-blurred images. In Experiment 2, pre-training on blurred images in a basic-level categorization task transferred to and improved the learning of subordinate-level categories relative to pre-training with non-blurred images. Such results support the theory that poor spatial acuity at birth may benefit early category learning.

### Effect of blur is dependent on color and other model training parameters

While the benefit of image blurring was robust across several experimental conditions, several factors affected the degree to which blurring was beneficial. First, in both experiments, these benefits only manifested when using grayscale images. This result suggests that the benefit of blurring for basic-level category learning is dependent on whether color is available as an additional cue to object category. Specifically, when the models we used have access to color information, they may learn at least partially based on color, thereby reducing the role of achromatic features (texture, shape). In contrast, when color information is not available in training images, our models necessarily rely more on achromatic features, including both fine-scale texture and coarse-scale shape information. However, in a color-free regime, the fine-scale details of images may be detrimental to learning basic-level categories. This is supported by past evidence that categorization in CNNs relies more on fine-scale details such as texture and less on coarse-scale features such as shape [35, 36]. For these reasons, blurring inputs to emphasize coarse scale shape information may result in a larger benefit to category learning when color is absent than when it is present.

The dependence of our results on the absence of color also has implications for relating our models to the human visual system. Specifically, past research has indicated that, in addition to poor spatial acuity, human infants have relatively poor sensitivity to color for the first 4–6 months of life [15]. Thus, the grayscale image training regime may be a better approximation of the environment experienced by infants early in development. The fact that we observe a benefit from blurring in this ecologically realistic setting, but not a less realistic setting, supports the idea that blurring confers a benefit in the context of the overall environment in which the infant visual system is situated. An important caveat to this is that, as mentioned previously, color vision matures more rapidly than visual acuity, meaning that color may quickly come to play some role in object categorization during development [18, 20]. Therefore, in order to better simulate the learning environment of developing infants, future work should also model the maturational pattern of chromatic vision relative to acuity.

We also note that the benefits of training with blurred images were only apparent when using specific hyperparameters and model training conditions. Using a learning rate scheduler (which sets the step size for weight updates) in Experiment 1 rendered the effect of blur less apparent as compared to when the scheduler was removed. We speculate that using the scheduler may have optimized learning. This rapid shift to extremely high performance may have yielded an environment in which there was little room for reduced input complexity to further improve performance. In contrast, infant categorization performance is quite poor and only slowly improves over many years to attain adult-like levels [37, 38]; see later subsections for more discussion of this point. One interesting possibility that should be pursued in future work is whether models using a learning rate scheduler, despite high performance, ultimately learn less robust basic-level category representations.

Another model parameter that had an impact on performance was the rate of decrease in blur: this manifested as an advantage for the linear-blur condition over the nonlinear-blur condition. In the former condition, the degree of blur (i.e., standard deviation of Gaussian filter) decreased linearly over time, while in the latter condition, the amount of blur decreased more rapidly at the start of training. Given that the standard deviation of the filter in the spatial domain is nonlinearly related to its standard deviation in the frequency domain, the nonlinear-blur condition actually may more closely approximate a linear increase in acuity than does the linear-blur condition. Based on the finding that acuity increases approximately linearly across human visual development [39], we had initially hypothesized that our nonlinear-blur models would achieve higher performance than our linear-blur models. However, our results showed the opposite. There are multiple plausible explanations for such a result. First, CNNs may benefit more from a linear time course of blur reduction because neural networks are likely sensitive to somewhat different learning parameters than humans. Second, it is also possible that our implementation of nonlinear blur was too fast relative to the time span over which we implemented blurring of images. Across several preliminary analyses, we found that our slowest nonlinear function (with a base of 2) was the most effective out of the tested nonlinear functions. Given the brief span in which the model was trained on blurred images, it is possible that the decrease in blur was still not gradual enough to allow the network to benefit from the reduction in input complexity afforded by blurring. Future work should explore models with more gradual nonlinear blurring functions as well as training on blurred images for longer periods of time in order to evaluate how these parameters affect performance in nonlinear models.

### Reduced input complexity enhances subordinate-level category learning

Somewhat unexpectedly, the results of Experiment 2 suggest that the benefit of early visual experience with blurred images generalizes to subordinate-level category learning. Models initially trained to perform basic-level categorization on ecoset showed their largest boost in ImageNet image classification accuracy when the initial training phase included blurred images. This boost was observed for both basic-level labeled images *and* subordinate-level labeled images from ImageNet. Given that subordinate-level categories are defined more by fine-grained shape and surface appearance [4], why does a model trained on a basic-level classification task for blurred images facilitate learning subordinate-level categories? One possibility is that there is some similarity in the representations needed for basic-level versus subordinate-level classification in that subordinate-level categories are refinements of basic-level categories. To the extent that this is true (e.g., most dog breeds are all visually recognizable as dogs), the basic-level representations learned by the linear-blur and nonlinear-blur models in Experiment 1 can be efficiently fine-tuned for a new subordinate-level task in Experiment 2. However, in the case of basic-level categories with subordinate-level members that deviate from the general category appearance (e.g., penguins and ostriches do not look like the majority of birds), our expectation is that fine-tuning for such subordinate-level categories will not benefit from pre-training with blurred images. Future work should include fine-grained analyses of category learning that take into account the specific visual and semantic structures of individual categories.

Related to the finding that basic-level pre-training benefits subsequent learning of subordinate-level categories, a recent study found that training a visual CNN model with *superordinate*-level labeled images (e.g., fruit, animal) followed by basic-level training resulted in a model that was highly robust to image perturbations, as well as exhibiting a stronger shape bias as compared to models trained without hierarchical labels [40]. In tandem with our results, such findings highlight the importance of considering the role of different hierarchical levels when creating training paradigms for neural networks. For example, ImageNet includes a mixture of basic-level and subordinate-level labels that are not meaningfully differentiated, thereby confounding two levels of category learning that are likely to be supported by different visual features. At the same time, some ImageNet categories labeled at the subordinate-level may effectively function as basic-level categories in that no other subordinate-level categories for their parent basic-level category are included in ImageNet. Within the work we present here, in Experiment 1 we controlled for this issue by using the ecoset dataset [28] which contains only basic-level labels and is motivated by the foundational role that the basic level plays in category learning and adult cognition [4]. Moreover, when using ImageNet in Experiment 2, we explicitly divided our results by basic- and subordinate-level labeled images. Future work should explore including superordinate-level labeled images to investigate how early experience with blurred inputs interacts with a hierarchical superordinate-to-basic training regime similar to that used by Ahn and colleagues [40].

### Limitations of the analogy between neural networks and human vision

While CNNs have many practical advantages that make them a useful model system for visual neuroscience, there are multiple ways in which these models are likely to diverge from biological visual systems. For example, as mentioned previously, as compared to humans, CNNs rely more strongly on local texture features than global shape [35, 36]. Relatedly, Baker et al. [41] evaluated a trained CNN with outlines of 2D objects and animals with varying internal patterns and found that CNNs often classify images based on texture and local shape rather than global shape. Moreover, model performance changed dramatically based on the color and texture inside the outlined objects.

Another point of divergence between CNNs and humans is the lack of generalization to new viewing conditions in terms of lighting, rotation, viewpoint, etc. Whereas humans are able to generalize to novel viewing conditions, several studies have found that CNNs tend to overfit to the conditions on which they are trained on and have difficulty adapting to new conditions. For example, Szegedy et al. [42] found that CNNs classified images differently when minimal changes, imperceptible to humans, were applied to the image. More generally, Serre [26] makes a strong case for CNNs serving as incomplete models of human vision: he reviews a variety of different human visual recognition behaviors that are poorly captured by CNNSs (e.g., perceptual grouping) and, similar to Szegedy et al. [42], multiple examples of failures to generalize due to the extreme sensitivity of CNNs to small noise perturbations. At the same time, O’Toole and Castillo [43] make a cogent argument for why CNNs are appropriate models for studying functional aspects of human vision and, in particular, for testing sophisticated hypotheses on human learning across the lifespan.

Another important aspect in which CNNs diverge from human learners is that CNN models are fully supervised: every input has a ground truth label that is used to train the network. In contrast, human infants appear to learn without supervision. For example, Eimas and Quinn [37] found that very young infants form categorical representations across a range of pairwise or multiway contrastive comparisons (e.g., cats vs. horses; or horses vs. cats, zebras, or giraffes) and that humans learn to further differentiate between visually-similar categories relatively early in development. Such findings suggest that human infants are able—almost entirely on the basis of visual inputs alone—to learn visually-based basic-level categories. Similarly, Quinn and Eimas [38] argue that the acquisition of conceptually-based representations is a gradual process which is bootstrapped by early, purely perceptual learning. Under this view, human learners scaffold their conceptual knowledge on the basis of a perceptually anchored structure that is learned in a largely unsupervised manner. In this regard, the use of CNNs to study human learning is not perfectly aligned.

Despite this potential difference, we argue that several factors support the use of supervised learning models in the context of our current study. First, there is increasing evidence that even very young children are self-supervising as part of learning visual categories [44]. Second, even at the earliest stages of development, caregivers provide linguistic supervision to human infants—such sparse supervision can be nearly as or even more effective (because feedback is provided for the most salient examples) than full supervision. Third, in our present study we focus on how reducing the complexity of visual inputs impacts the rate and ultimate accuracy of visual classification; while supervision might improve overall performance relative to an unsupervised system, it is less likely to interact with input manipulations. If anything, we predict that an unsupervised learner would receive *greater* benefit from a reduction in input complexity in that the dimensionality of the visual similarity space is reduced. As a consequence, within-category objects appear more similar to one another and are more readily learned as members of the same basic-level category. In this light, future work should examine how low-pass filtering affects learning in unsupervised models of visual classification.

Further supporting the validity of comparisons across artificial neural networks and biological systems is the longstanding finding that goal-driven CNNs trained on an object categorization task common to human visual behavior learn object representations quite similar to neural representations of the same objects [25]. More generally, a wide array of studies have found that CNNs are able to account for much of the neural response variance in object viewing tasks as measured by fMRI in humans or by neurophysiological recordings in monkeys [12, 45, 46]. Given these representational similarities, CNNs provide an experimental setting in which to explore, using high-performing models and complex, real-world images, how learning is affected by the manipulation of a wide range of visual attributes and learning conditions.

### Relation to previous work

As discussed earlier, several recent studies similar to ours have investigated how training with blurred images impacts learning in neural network models [30–33]. However, the central task we explored—basic-level object categorization—was not observed to benefit from low-pass image training in any of these reports. Indeed, contrary to our results, Avberšek et al. [30] reported that, for categorization of unblurred images, models trained on intact images performed better than models trained on blurred images. Similarly, Jang and Tong [32] did not observe an overall benefit in object categorization for models trained on blurred images as compared to models trained on unblurred images.

There are several reasons for the discrepancy between these studies and ours. First, we utilized the ecoset database, which includes only basic-level categories, while these prior studies used the mixed category ImageNet database. Second, initial training with low-pass inputs only affected performance using grayscale images, while some of these studies used colored images as inputs. Third, some of these studies focused on the robustness of their models to blurred images but did not examine the trajectory of learning or ultimate performance across different conditions. Finally, other factors such as network architecture and training parameters may also contribute to differences between our present study and earlier studies. Perhaps most critically, the *functional* parameters we adopted were specifically targeted at mirroring the learning environment of the human infant: reducing image resolution at initial learning, increasing resolution only slowly over learning, removing color, and restricting the task to basic-level category learning. Under these specific conditions, we find a clear benefit for models whose training includes blurred inputs at initial learning.

### Changes in conceptual object representations across development

Our results are consistent with developmental evidence for a change in how basic-level categories are represented across development. Jüttner et al. [47] found that younger children are able to perform object discriminations relying on object part structure but fail on tasks that require metric part relations. In contrast, older, school-aged children are able to make metric discriminations between visual categories. As such, the visual discrimination trajectory articulated in Jüttner et al.’s study reflects a developmental trajectory in which coarser object part structures are available in the low-pass filtered images available early in development, but that metric relations only become available as more fine-grained resolution inputs come into play with the maturation of the visual system. This change in conceptual learning reinforces our point that the developmental path of visual acuity is central to bootstrapping both initial basic-level category learning and later general category learning—at which point, a wide variety of information sources, including part structure, metric part relations, linguistic feedback, semantics, affordances, etc. may participate in refining and enhancing the core organization of knowledge built on visual similarity. Future work should explore model behavior across the different perceptual tasks that map human development as well as how this wider range of factors interact with category learning within these models (e.g., [12]).

## Conclusion

In conclusion, our simulations support the hypothesis that low visual acuity in early development (as a consequence of low contrast sensitivity) may be a key factor in infant visual growth and cognitive development, providing an early advantage in basic-level category learning. Although blurry inputs were only presented briefly at the start of training, early performance advantages were sustained throughout the duration of basic-level training and, as established in separate experiments, persisted through the introduction of subordinate-level categorization tasks. Thus, poor vision early in life, rather than hindering learning, may be, in part, a functional adaptation that supports the human infant’s acquisition of robust conceptual structures.

From a broader perspective, this conclusion highlights the ways in which near-term developmental benefits may emerge as a consequence of the human infant’s underdeveloped cognitive and perceptual mechanisms [3]. At the same time, there may be long-term benefits to prematurity at birth. For example, Piantadosi and Kidd [2] speculate that a positive feedback loop has selected for intelligence whereby human infants’ premature state at birth enables the development of larger brains (and greater intelligence), but this same helplessness leads to a need for superior intelligence in adult caregivers. More generally, we suggest that while there may be evolutionary-scale benefits for the altricial state of human newborns, more specific selective pressures tied to perceptual or cognitive capacities also contribute to the premature state of human newborns.

Reinforcing this claim, our findings are consistent with other recent studies indicating that the trajectory of human visual development has positive functional implications [8, 31, 32]. This body of research illustrates how a reduction in input complexity in early development [21, 48], as well as the overall trajectory of development [3], can help facilitate learning within a variety of domains. Of note, a parallel approach for training models—referred to as *curriculum learning*—has become popular in the machine learning community (similarly inspired by Elman’s 1993 paper [21]). As articulated by Bengio et al. [49], more effective learning can be facilitated when inputs are ordered with a gradual increase in both number and complexity.

Given this growing interest in how the organization of inputs can influence learning, future work should explore how developmental trajectories across all perceptual systems may impact the acquisition of adult-like abilities. With respect to the specific question of category acquisition, we see several fruitful areas of potential investigation. First, our results suggest that the organization of early experience impacts category learning at multiple hierarchical levels. Thus, further experiments with better curated multi-level category datasets should be used to explore how different modes of complexity reduction may facilitate learning across categorical hierarchies. Second, the specific roles of visual attributes, including color, spatial frequency, contrast, acuity, and foveation should be explored with respect to their general effect on category learning, as well as how these attributes might interact with the acquisition of specific categories that are defined more or less by particular visual properties. Third, while early category learning may be strongly driven by visual inputs, supervision—in the form of category labels via speech, environmental sounds [50], etc.—may, sparsely at least, provide learners with important information about category membership. Following Stretcu et al.’s [51] demonstration that a coarse to fine approach to training improves classification performance, state-of-the-art multimodal classification models [52] should be used to examine whether reducing input complexity yields equal benefits across modalities. While even fully supervised models may show a benefit to using a curriculum learning training strategy, because a reduction in input complexity allows the learner to better align representations across modalities, we predict much larger benefits in category learning when using sparsely supervised or unsupervised models. Exploring these and related questions will enable a better understanding of how the structure of our environment influences the emergence of high-level conceptual knowledge structures in human development.

## Materials and methods

Our study is split into two distinct sections. First, Experiment 1, using standard CNNs, explores whether using blurred visual inputs during the initial learning of basic-level categories impact model accuracy and/or rate of learning. Second, Experiment 2, using the same CNNs trained in Experiment 1 which are fine-tuned on a new task, explores whether the basic-level representations learned with initially blurred visual inputs also confer benefits for learning of subordinate-level categories.

### Models and general training procedures

For all models in both experiments, we used the ResNet-50 architecture [29] with a learning rate of 0.1 and a standard gradient descent (SGD) optimizer. ResNet-50 is a type of deep neural network model which has 50 total layers (48 convolutional layers, 1 maxpool layer, and 1 average pool layer). For models trained on color images, three different inputs representing the RGB values of each pixel in each image were used. For models trained on grayscale images, a single input representing the brightness of each pixel in each image was used. Models were trained using PyTorch version 1.10.0 in Python version 3.7.1, on the Carnegie Mellon Neuroscience Institute High Performance Computing Cluster which consists of 21 CPU nodes and 12 GPU nodes, 280TB terabytes of shared disk space and 2.8 terabytes of RAM (https://ni.cmu.edu/computing/knowledge-base/computing-facilities-description-overview/). For our SGD optimizer, we set momentum to 0.9 and weight decay to 0.1. No learning rate scheduler was used, a decision motivated by our finding in initial tests that when a scheduler was used, all of our models performed similarly regardless of blur condition; see *Discussion*. Individual models were each trained for 300 epochs (Exp. 1) or 150 epochs (Exp. 2).

### Image datasets

For Experiment 1, model training was performed using the ecoset image dataset, which contains over 1.5 million images drawn from 565 labeled basic-level categories [28]. This dataset contains only basic-level categories that have clear, commonly used basic-level names, for example, “table”, “dog”, or “cat”. For Experiment 2, we used the ImageNet dataset [27]. ImageNet is more commonly used in computer vision research, and contains 1000 categories, including a mix of basic- and subordinate-level labels. Multiple categories included in ImageNet are subordinate to the basic-level categories in ecoset (i.e., numerous bird species or dog breeds). The inclusion of both basic and subordinate-level categories in ImageNet was our motivation for using it in Experiment 2. To control for the fact that the images in ecoset and ImageNet were variable in size, we used the PIL image processing library in Python to resize each image by center cropping based on the minimum dimension between width and height and then resizing all images to 224x224 pixels (code available at https://github.com/tarrlab/startingblurry).

### Experiment 1 training procedure

To explore the impact of blurring during learning, we trained multiple models on image sets defined by different numbers of blurred and non-blurred images presented at the beginning of training. For each epoch, 50,000 images were randomly selected from the training dataset. All models were trained on the same randomly selected images at each epoch, but with different amounts of blur applied to the training images during pre-processing. The use of 50,000 training images per epoch was selected based on a balance between having a sufficient number of images to adequately train the model and limiting the number of images to allow gradual blur to have some impact on learning. For all blurred models, images were blurred for only the first 50 epochs (i.e., 2,500,000 images), although the exact time course of blur reduction differed between the different blur conditions (see *Implementation of image blur during model training)*. Pilot testing using blurring for the first 50, 100, 150, 200, 250, or 300 epochs in different models revealed that limiting blur to the beginning of training—50 epochs—produced our most robust results. To compute validation accuracy at each epoch, we matched the blur applied to the validation image dataset to the degree of blur applied to the training images for that epoch, ran the blurred validation images through our model, and then calculated the number of images labeled correctly relative to the total number of images in the validation dataset.

### Implementation of image blur during model training

Blurring was implemented using a Gaussian filter via the GaussianBlur function in the transformations module under Torchvision (a Pytorch library). The GaussianBlur filter applies a low-pass filter to each image by removing any spatial frequencies finer than the scale of the Gaussian (Fig 1C and 1D). Implementing a Gaussian blur filter requires specifying a sigma (σ, which denotes the standard deviation of the Gaussian filter, in pixels) and a kernel size value based on the epoch. In all blurred models, the standard deviation was initially σ = 5 and was reduced until the 50th training epoch, at which point σ = 0.25. Our implementation and manipulation of Gaussian blur is similar to past work that examined how image blur impacts different aspects of visual learning [31, 32].

In different model training conditions, we used either a linear or a logarithmic function to determine the degree to which σ was reduced in each subsequent epoch. The linear function (*linear-blur* condition) was defined by calculating the difference between our initial and final σ values and dividing by the number of epochs in which images were blurred (50). The value of σ was then decreased by this increment following each epoch. The logarithmic function (*nonlinear-blur* condition) was defined by reducing the value of σ according to a logarithmic function with a base of 2, which results in a decrease in σ that is of greater magnitude at the beginning of training (Fig 1B). Since the σ of our Gaussian kernel is related nonlinearly to the resulting low-pass frequency cutoff of the image (our approximation of visual acuity), a logarithmic change in σ means that the change in acuity over time will more closely approximate a linear function—similar to that measured in human development [39]. For both functions, kernel size was calculated as 8 times the σ value plus 1 to ensure that: 1) the kernel size was an odd number; 2) the entire kernel was sufficiently large so as to accommodate ±4 standard deviations from the center of the filter.

### Color manipulations

In addition to the amount of blur applied to each image, we also manipulated the color content of images by using either color or grayscale images. Our rationale for manipulating color was two-fold. First, poor contrast sensitivity in infants also limits their ability to see color differences [13]. Thus, images absent color in addition to image blur may better approximate a human infant’s visual experience in the early months of their development. Second, while color can play a role in human categorization [20, 34], color is not consistent or diagnostic for many basic-level categories. To the extent that CNNs tend to overfit, fine-grained details such as color or texture may support category learning at the expense of more general shape properties [35]. Models trained with grayscale images were expected to have a stronger shape bias and, consequently, to allow for a larger impact of spatial frequency manipulations during learning.

### Experiment 1 model conditions

We implemented six total model conditions. These consisted of three blur conditions: *non-blurred* (normal unfiltered inputs throughout training), *linear-blur* (a linear decrease in blur over the first 50 training epochs), and *nonlinear-blur* (a logarithmic decrease in blur over the first 50 training epochs). These were crossed with two color conditions: *color* and *grayscale* images. For each of these six conditions, we ran 10 replications (which we refer to as trials) using different random seeds with otherwise identical models. To ensure comparability of our results across conditions, we used the same randomly selected ecoset images for each epoch during training and used the complete validation set after each training epoch (although the appearance of each image in terms of blur and color varied with the condition).

### Experiment 1 data analysis

For the purpose of visualization and statistical analyses, we temporally smoothed the validation accuracy results for each individual model by computing the moving average over a sliding window of 20 epochs. All subsequent statistical analyses were performed on this smoothed data. Learning rate was estimated by finding the difference in validation accuracy between neighboring epochs (i.e., the approximate slope of the accuracy), and then applying a second moving average filter with a window size of 20 epochs. For both the accuracy and learning rate, we then tested for significant differences between conditions at each time point using a linear mixed effects model, implemented using the Python package *statsmodels*. Across a sliding window where 20 epochs were considered at a time, we constructed a model where Condition and Epoch number were fixed effects (categorical and continuous, respectively) and the Trial Number was the random effect. This analysis provides a coefficient and *p*-value for the effect of Condition, for each possible pairwise condition comparison (non-blurred vs. linear-blur, non-blurred vs. nonlinear-blur, linear-blur vs. nonlinear-blur). Comparisons were always made between different blur conditions within the same color condition only. Finally, the resulting *p*-values from all pairwise comparisons, as shown in Fig 2, were FDR corrected across all epochs using the Benjamini-Hochberg procedure implemented in *statsmodels*, with ɑ = 0.05 [53]. In addition to this sliding window procedure, we computed the time-averaged validation accuracy for each of the six model conditions and performed a two-way ANOVA on these values with factors of *Color* and *Blur*.

### Experiment 2 training procedure

To explore whether the basic-level categorization results also generalized to subordinate-level classification, we used a transfer learning paradigm in which the trained models from Experiment 1 were fine-tuned using ImageNet [27]. Beyond this shift in the image dataset, Experiment 2 used the same overall training procedure as Experiments 1. All models were fine-tuned using the same 50,000 randomly selected images (now from ImageNet) for each epoch, and images were resized using the same center crop method as used in Experiment 1. Color content was held constant from ecoset to ImageNet: when using an ecoset pre-trained model that was initially trained with grayscale images, all ImageNet fine-tuning was performed with grayscale images, and vice versa for color models. To maintain the same learning environment as in Experiment 1, models in Experiment 2 used the same architecture and hyperparameters, except for two differences. First, because Experiment 2 is based on fine tuning (and not training from scratch), models were fine-tuned for only 150 epochs (rather than 300 epochs). Second, because of the larger number of classes labeled in ImageNet, models used for fine-tuning had a final layer with 1,000 units (rather than 565 units).

### Pre-trained model selection

To determine which models would serve as the base for Experiment 2, we identified the best training time point (based on validation accuracy) for each model from Experiment 1, and the weights from the model at this time point were stored (note that this could be a different time point for different trials within a given condition). These stored models were each fine-tuned with ImageNet images across 150 epochs. In addition to these stored models, we also trained, from scratch with random initial weights, two sets of control models with either grayscale or color images. This resulted in eight model conditions in total—six pre-trained model conditions and two control model conditions. As in Experiment 1, we ran 10 trials for each condition. For each trial within each pre-trained model condition, the pre-trained models were from the corresponding trials in Experiment 1. For example, during Trial 4 of Experiment 2, the starting point in each condition was Trial 4 of the corresponding model from that same condition from Experiment 1.

### Experiment 2 data analysis

To calculate validation accuracy and learning rate, we used the same averaging techniques as in Experiment 1. Statistical tests comparing training conditions were also identical to the analyses used in Experiment 1. We also performed an analysis in which we computed the validation set accuracy separately for the ImageNet categories that were defined as basic-level versus those defined as subordinate-level. We focused on the models trained with grayscale images only because these models showed the largest benefit from blurring during training. ImageNet categories were defined as basic- or subordinate-level categories based on human annotations for how frequently a given category name was likely to be used in everyday language to refer to the object of interest [4]. For example, “bee” and “strawberry” were labeled as basic-level, while “Yorkshire terrier” and “Granny-smith apple” were labeled as subordinate-level. The full list of basic- and subordinate-level assignments for the 1000 ImageNet categories is available in our supplementary materials (S1 and S2 Tables).

To compute accuracy for the basic- and subordinate-level categories, we identified the best training time point with respect to validation accuracy for each individually fine-tuned model in Experiment 2. The weights at this time point were saved, and the entire ImageNet validation dataset was run through each model using these saved weights. Accuracy was computed for one category label at a time and was defined as the number of correctly classified images with that label divided by the total number of images with that label. The resulting category-specific accuracy values were then averaged over all basic-level categories or all subordinate-level categories. We also computed the overall validation accuracy of each model across all categories. In computing overall validation accuracy, we always used the same time point as was used to generate the basic- and subordinate-level accuracy values (i.e., the time point for each model with the single best validation accuracy value). Finally, we performed statistical comparisons between the four different pre-training conditions (no pre-training, pre-training with non-blurred images, pre-training with linear-blur images, pre-training with nonlinear-blur images) using two-tailed independent samples *t*-tests between each pair of conditions, implemented using the Python package *scipy*. The resulting *p*-values from all pairwise comparisons, as shown in Fig 4, were FDR corrected as described above.

## Supporting information

S1 Table ImageNet categories were manually labeled as either basic- or subordinate-level (see *Methods*); this table lists the English names of the categories labeled as basic- and subordinate-level. To find the folder ID-to-category name correspondence, see the file “bOrS.csv” in our Github repository (https://github.com/ojinsi/startingblurry).
(DOCX)

S2 Table ImageNet categories were manually labeled as either basic- or subordinate-level (see *Methods*); this table lists the ecoset folder IDs of the categories labeled as basic- and subordinate-level. To find the category-to-folder ID name correspondence, see the file “bOrS.csv” in our Github repository (https://github.com/ojinsi/startingblurry).
(DOCX)
