# Supplementary material for: Early experience with low-pass filtered images facilitates visual category learning in a neural network model
Source: PLoS One. 2023 Jan 6;18(1):e0280145. doi: 10.1371/journal.pone.0280145 (PMC9821476; doi:10.1371/journal.pone.0280145)
Supplement: S1 Table — To find the folder ID-to-category name correspondence, see the file “bOrS.csv” in our Github repository (https://github.com/ojinsi/startingblurry). (DOCX) [file pone.0280145.s001.docx]

**SI Table 1.** ImageNet categories were manually labeled as either basic- or subordinate-level (see *Methods*); this table lists the English names of the categories labeled as basic- and subordinate-level. To find the folder ID-to-category name correspondence, see the file “bOrS.csv” in our Github repository (<https://github.com/ojinsi/startingblurry>).

***Basic-level ImageNet category labels***

| cougar | gazelle | porcupine | sea_lion | badger |
| --- | --- | --- | --- | --- |
| killer_whale | mink | jaguar | hyena | meerkat |
| skunk | weasel | coyote | mongoose | tiger |
| zebra | ram | orangutan | leopard | chimpanzee |
| guinea_pig | gorilla | ox | hare | baboon |
| hog | snow_leopard | hamster | Tibetan_terrier | water_buffalo |
| bison | hippopotamus | giant_panda | armadillo | llama |
| lion | beaver | cheetah | otter | koala |
| echidna | wallaby | platypus | wombat | revolver |
| umbrella | schooner | soccer_ball | accordion | ant |
| starfish | chambered_nautilus | laptop | strawberry | airship |
| balloon | space_shuttle | gondola | canoe | catamaran |
| aircraft_carrier | submarine | tank | missile | bobsled |
| barrow | shopping_cart | motor_scooter | forklift | amphibian |
| ambulance | cab | jeep | limousine | minivan |
| Model_T | go-kart | golfcart | moped | snowplow |
| fire_engine | garbage_truck | pickup | tow_truck | trailer_truck |
| streetcar | snowmobile | tractor | mobile_home | tricycle |
| unicycle | bookcase | china_cabinet | medicine_chest | table_lamp |
| file | park_bench | barber_chair | throne | rocking_chair |
| studio_couch | toilet_seat | desk | pool_table | dining_table |
| entertainment_center | wardrobe | orange | lemon | fig |
| pineapple | banana | jackfruit | pomegranate | acorn |
| hip | ear | corn | buckeye | organ |
| drum | gong | maraca | marimba | banjo |
| cello | violin | harp | cornet | French_horn |
| trombone | harmonica | ocarina | panpipe | bassoon |
| oboe | sax | flute | cliff | valley |
| volcano | sandbar | coral_reef | seashore | geyser |
| hatchet | cleaver | letter_opener | plane | power_drill |
| lawn_mower | hammer | corkscrew | can_opener | plunger |
| screwdriver | shovel | plow | chain_saw | ostrich |
| king_penguin | barracouta | eel | common_iguana | Komodo_dragon |
| triceratops | African_crocodile | common_newt | whistle | wing |
| paintbrush | hand_blower | oxygen_mask | snorkel | loudspeaker |
| microphone | screen | mouse | electric_fan | oil_filter |
| strainer | space_heater | stove | guillotine | barometer |
| rule | odometer | scale | digital_clock | hourglass |
| sundial | parking_meter | stethoscope | syringe | magnetic_compass |
| binoculars | projector | sunglasses | loupe | radio_telescope |
| bow | cannon | assault_rifle | rifle | projectile |
| crane | lighter | abacus | cash_machine | slide_rule |
| desktop_computer | hand-held_computer | notebook | web_site | harvester |
| thresher | printer | slot | vending_machine | sewing_machine |
| joystick | switch | hook | car_wheel | paddlewheel |
| pinwheel | potter's_wheel | gas_pump | carousel | swing |
| reel | radiator | puck | hard_disc | sunglass |
| pick | car_mirror | solar_dish | remote_control | disk_brake |
| buckle | hair_slide | knot | combination_lock | padlock |
| nail | safety_pin | screw | muzzle | seat_belt |
| ski | candle | jack-o'-lantern | spotlight | torch |
| neck_brace | pier | tripod | maypole | mousetrap |
| spider_web | trilobite | harvestman | scorpion | tick |
| centipede | isopod | crayfish | hermit_crab | ladybug |
| weevil | fly | bee | grasshopper | cricket |
| walking_stick | cockroach | mantis | cicada | leafhopper |
| lacewing | dragonfly | damselfly | lycaenid | jellyfish |
| sea_anemone | brain_coral | flatworm | nematode | conch |
| snail | slug | sea_slug | chiton | sea_urchin |
| sea_cucumber | iron | espresso_maker | microwave | Dutch_oven |
| rotisserie | toaster | waffle_iron | vacuum | dishwasher |
| refrigerator | washer | Crock_Pot | frying_pan | wok |
| caldron | coffeepot | teapot | spatula | altar |
| triumphal_arch | patio | steel_arch_bridge | suspension_bridge | viaduct |
| barn | greenhouse | palace | monastery | library |
| apiary | boathouse | church | mosque | stupa |
| planetarium | restaurant | cinema | home_theater | lumbermill |
| coil | obelisk | totem_pole | castle | prison |
| grocery_store | bakery | barbershop | bookshop | butcher_shop |
| confectionery | shoe_shop | tobacco_shop | toyshop | fountain |
| cliff_dwelling | yurt | dock | brass | megalith |
| banister | breakwater | dam | stone_wall | grille |
| sliding_door | turnstile | mountain_tent | scoreboard | honeycomb |
| plate_rack | pedestal | beacon | mashed_potato | bell_pepper |
| head_cabbage | broccoli | cauliflower | zucchini | cucumber |
| artichoke | cardoon | mushroom | shower_curtain | jean |
| carton | handkerchief | sandal | ashcan | safe |
| plate | necklace | croquet_ball | fur_coat | thimble |
| pajama | running_shoe | cocktail_shaker | chest | manhole_cover |
| modem | tub | tray | balance_beam | bagel |
| prayer_rug | kimono | hot_pot | whiskey_jug | knee_pad |
| book_jacket | spindle | ski_mask | beer_bottle | crash_helmet |
| bottlecap | tile_roof | mask | maillot | Petri_dish |
| football_helmet | bathing_cap | teddy | holster | pop_bottle |
| photocopier | vestment | crossword_puzzle | golf_ball | trifle |
| suit | water_tower | feather_boa | cloak | drumstick |
| shield | Christmas_stocking | hoopskirt | menu | stage |
| bonnet | meat_loaf | baseball | face_powder | scabbard |
| sunscreen | beer_glass | hen-of-the-woods | guacamole | lampshade |
| wool | hay | bow_tie | mailbag | water_jug |
| bucket | dishrag | soup_bowl | eggnog | mortar |
| trench_coat | paddle | chain | swab | mixing_bowl |
| potpie | wine_bottle | shoji | bulletproof_vest | drilling_platform |
| binder | cardigan | sweatshirt | pot | birdhouse |
| hamper | ping-pong_ball | pencil_box | pay-phone | consomme |
| apron | punching_bag | backpack | groom | bearskin |
| pencil_sharpener | broom | mosquito_net | abaya | mortarboard |
| poncho | crutch | Polaroid_camera | space_bar | cup |
| racket | traffic_light | quill | radio | dough |
| cuirass | military_uniform | lipstick | shower_cap | monitor |
| oscilloscope | mitten | brassiere | French_loaf | vase |
| milk_can | rugby_ball | paper_towel | earthstar | envelope |
| miniskirt | cowboy_hat | trolleybus | perfume | bathtub |
| hotdog | coral_fungus | bullet_train | pillow | toilet_tissue |
| cassette | carpenter's_kit | ladle | stinkhorn | lotion |
| hair_spray | academic_gown | dome | crate | wig |
| burrito | pill_bottle | chain_mail | theater_curtain | window_shade |
| barrel | washbasin | ballpoint | basketball | bath_towel |
| cowboy_boot | gown | window_screen | agaric | cellular_telephone |
| nipple | barbell | mailbox | lab_coat | fire_screen |
| minibus | packet | maze | pole | horizontal_bar |
| sombrero | pickelhaube | rain_barrel | wallet | cassette_player |
| comic_book | piggy_bank | street_sign | bell_cote | fountain_pen |
| Windsor_tie | volleyball | overskirt | sarong | purse |
| bolo_tie | bib | parachute | sleeping_bag | television |
| swimming_trunks | measuring_cup | espresso | pizza | breastplate |
| shopping_basket | wooden_spoon | saltshaker | chocolate_sauce | ballplayer |
| goblet | gyromitra | stretcher | water_bottle | dial_telephone |
| soap_dispenser | jersey | school_bus | jigsaw_puzzle | plastic_bag |
| reflex_camera | diaper | Band_Aid | ice_lolly | velvet |
| tennis_ball | gasmask | doormat | Loafer | ice_cream |
| pretzel | quilt | maillot | tape_player | clog |
| iPod | bolete | scuba_diver | pitcher | matchstick |
| bikini | sock | CD_player | lens_cap | thatch |
| vault | beaker | bubble | cheeseburger | parallel_bars |
| flagpole | coffee_mug | rubber_eraser | stole | carbonara |
| dumbbell |  |  |  |  |

***Subordinate-level ImageNet category labels***

| kit_fox | English_setter | Siberian_husky | Australian_terrier | English_springer |
| --- | --- | --- | --- | --- |
| grey_whale | lesser_panda | Egyptian_cat | ibex | Persian_cat |
| malamute | Great_Dane | Walker_hound | Welsh_springer_spani | whippet |
| Scottish_deerhound | African_elephant | Weimaraner | soft-coated_wheaten_ | Dandie_Dinmont |
| red_wolf | Old_English_sheepdog | otterhound | bloodhound | Airedale |
| giant_schnauzer | titi | three-toed_sloth | sorrel | black-footed_ferret |
| dalmatian | black-and-tan_coonho | papillon | Staffordshire_bullte | Mexican_hairless |
| Bouvier_des_Flandres | miniature_poodle | Cardigan | malinois | bighorn |
| fox_squirrel | colobus | tiger_cat | Lhasa | impala |
| Yorkshire_terrier | Newfoundland | brown_bear | red_fox | Norwegian_elkhound |
| Rottweiler | hartebeest | Saluki | grey_fox | schipperke |
| Pekinese | Brabancon_griffon | West_Highland_white_ | Sealyham_terrier | guenon |
| indri | Irish_wolfhound | wild_boar | EntleBucher | French_bulldog |
| basenji | Bernese_mountain_dog | Maltese_dog | Norfolk_terrier | toy_terrier |
| vizsla | cairn | squirrel_monkey | groenendael | clumber |
| Siamese_cat | komondor | Afghan_hound | Japanese_spaniel | proboscis_monkey |
| white_wolf | ice_bear | borzoi | toy_poodle | Kerry_blue_terrier |
| Scotch_terrier | Tibetan_mastiff | spider_monkey | Doberman | Boston_bull |
| Greater_Swiss_Mounta | Appenzeller | Shih-Tzu | Irish_water_spaniel | Pomeranian |
| Bedlington_terrier | warthog | Arabian_camel | siamang | miniature_schnauzer |
| collie | golden_retriever | Irish_terrier | affenpinscher | Border_collie |
| boxer | silky_terrier | beagle | Leonberg | German_short-haired_ |
| patas | dhole | macaque | Chesapeake_Bay_retri | bull_mastiff |
| kuvasz | capuchin | pug | curly-coated_retriev | Norwich_terrier |
| flat-coated_retrieve | keeshond | Eskimo_dog | Brittany_spaniel | standard_poodle |
| Lakeland_terrier | Gordon_setter | dingo | standard_schnauzer | Arctic_fox |
| wire-haired_fox_terr | basset | American_black_bear | Angora | howler_monkey |
| chow | American_Staffordshi | Shetland_sheepdog | Great_Pyrenees | Chihuahua |
| tabby | marmoset | Labrador_retriever | Saint_Bernard | Samoyed |
| bluetick | redbone | polecat | marmot | kelpie |
| gibbon | miniature_pinscher | wood_rabbit | Italian_greyhound | cocker_spaniel |
| Irish_setter | dugong | Indian_elephant | Sussex_spaniel | Pembroke |
| Blenheim_spaniel | Madagascar_cat | Rhodesian_ridgeback | lynx | African_hunting_dog |
| langur | Ibizan_hound | timber_wolf | English_foxhound | briard |
| sloth_bear | Border_terrier | German_shepherd | tusker | grand_piano |
| airliner | warplane | fireboat | speedboat | lifeboat |
| yawl | trimaran | container_ship | liner | pirate |
| wreck | half_track | dogsled | bicycle-built-for-tw | mountain_bike |
| freight_car | passenger_car | electric_locomotive | steam_locomotive | beach_wagon |
| convertible | racer | sports_car | moving_van | police_van |
| recreational_vehicle | horse_cart | jinrikisha | oxcart | bassinet |
| cradle | crib | four-poster | chiffonier | folding_chair |
| Granny_Smith | custard_apple | rapeseed | upright | chime |
| steel_drum | acoustic_guitar | electric_guitar | daisy | yellow_lady's_slippe |
| alp | promontory | lakeside | cock | hen |
| brambling | goldfinch | house_finch | junco | indigo_bunting |
| robin | bulbul | jay | magpie | chickadee |
| water_ouzel | kite | bald_eagle | vulture | great_grey_owl |
| black_grouse | ptarmigan | ruffed_grouse | prairie_chicken | peacock |
| quail | partridge | African_grey | macaw | sulphur-crested_cock |
| lorikeet | coucal | bee_eater | hornbill | hummingbird |
| jacamar | toucan | drake | red-breasted_mergans | goose |
| black_swan | white_stork | black_stork | spoonbill | flamingo |
| American_egret | little_blue_heron | bittern | crane | limpkin |
| American_coot | bustard | ruddy_turnstone | red-backed_sandpiper | redshank |
| dowitcher | oystercatcher | European_gallinule | pelican | albatross |
| great_white_shark | tiger_shark | hammerhead | electric_ray | stingray |
| coho | tench | goldfish | rock_beauty | anemone_fish |
| lionfish | puffer | sturgeon | gar | loggerhead |
| leatherback_turtle | mud_turtle | terrapin | box_turtle | banded_gecko |
| American_chameleon | whiptail | agama | frilled_lizard | alligator_lizard |
| Gila_monster | green_lizard | African_chameleon | American_alligator | thunder_snake |
| ringneck_snake | hognose_snake | green_snake | king_snake | garter_snake |
| water_snake | vine_snake | night_snake | boa_constrictor | rock_python |
| Indian_cobra | green_mamba | sea_snake | horned_viper | diamondback |
| sidewinder | European_fire_salama | eft | spotted_salamander | axolotl |
| bullfrog | tree_frog | tailed_frog | analog_clock | wall_clock |
| stopwatch | digital_watch | computer_keyboard | typewriter_keyboard | black_and_gold_garde |
| barn_spider | garden_spider | black_widow | tarantula | wolf_spider |
| Dungeness_crab | rock_crab | fiddler_crab | king_crab | American_lobster |
| spiny_lobster | tiger_beetle | ground_beetle | long-horned_beetle | leaf_beetle |
| dung_beetle | rhinoceros_beetle | admiral | ringlet | monarch |
| cabbage_butterfly | sulphur_butterfly | chainlink_fence | picket_fence | worm_fence |
| spaghetti_squash | acorn_squash | butternut_squash | red_wine |  |
