# Supplementary material for: Early experience with low-pass filtered images facilitates visual category learning in a neural network model
Source: PLoS One. 2023 Jan 6;18(1):e0280145. doi: 10.1371/journal.pone.0280145 (PMC9821476; doi:10.1371/journal.pone.0280145)
Supplement: S2 Table — To find the category-to-folder ID name correspondence, see the file “bOrS.csv” in our Github repository (https://github.com/ojinsi/startingblurry). (DOCX) [file pone.0280145.s002.docx]

**SI Table 2.** ImageNet categories were manually labeled as either basic- or subordinate-level (see *Methods*); this table lists the ecoset folder IDs of the categories labeled as basic- and subordinate-level. To find the category-to-folder ID name correspondence, see the file “bOrS.csv” in our Github repository (<https://github.com/ojinsi/startingblurry>).

***Basic-Level Category ImageNet Folder IDs***

| n02125311 | n02423022 | n02346627 | n02077923 | n02447366 | n02071294 | n02442845 | n02128925 | n02117135 | n02138441 |
| --- | --- | --- | --- | --- | --- | --- | --- | --- | --- |
| n02445715 | n02441942 | n02114855 | n02137549 | n02129604 | n02391049 | n02412080 | n02480495 | n02128385 | n02481823 |
| n02364673 | n02480855 | n02403003 | n02326432 | n02486410 | n02395406 | n02128757 | n02342885 | n02097474 | n02408429 |
| n02410509 | n02398521 | n02510455 | n02454379 | n02437616 | n02129165 | n02363005 | n02130308 | n02444819 | n01882714 |
| n01872401 | n01877812 | n01873310 | n01883070 | n04086273 | n04507155 | n04147183 | n04254680 | n02672831 | n02219486 |
| n02317335 | n01968897 | n03642806 | n07745940 | n02692877 | n02782093 | n04266014 | n03447447 | n02951358 | n02981792 |
| n02687172 | n04347754 | n04389033 | n03773504 | n02860847 | n02797295 | n04204347 | n03791053 | n03384352 | n02704792 |
| n02701002 | n02930766 | n03594945 | n03670208 | n03770679 | n03777568 | n03444034 | n03445924 | n03785016 | n04252225 |
| n03345487 | n03417042 | n03930630 | n04461696 | n04467665 | n04335435 | n04252077 | n04465501 | n03776460 | n04482393 |
| n04509417 | n02870880 | n03018349 | n03742115 | n04380533 | n03337140 | n03891251 | n02791124 | n04429376 | n04099969 |
| n04344873 | n04447861 | n03179701 | n03982430 | n03201208 | n03290653 | n04550184 | n07747607 | n07749582 | n07753113 |
| n07753275 | n07753592 | n07754684 | n07768694 | n12267677 | n12620546 | n13133613 | n12144580 | n12768682 | n03854065 |
| n03249569 | n03447721 | n03720891 | n03721384 | n02787622 | n02992211 | n04536866 | n03495258 | n03110669 | n03394916 |
| n04487394 | n03494278 | n03840681 | n03884397 | n02804610 | n03838899 | n04141076 | n03372029 | n09246464 | n09468604 |
| n09472597 | n09421951 | n09256479 | n09428293 | n09288635 | n03498962 | n03041632 | n03658185 | n03954731 | n03995372 |
| n03649909 | n03481172 | n03109150 | n02951585 | n03970156 | n04154565 | n04208210 | n03967562 | n03000684 | n01518878 |
| n02056570 | n02514041 | n02526121 | n01677366 | n01695060 | n01704323 | n01697457 | n01630670 | n04579432 | n04592741 |
| n03876231 | n03483316 | n03868863 | n04251144 | n03691459 | n03759954 | n04152593 | n03793489 | n03271574 | n03843555 |
| n04332243 | n04265275 | n04330267 | n03467068 | n02794156 | n04118776 | n03841143 | n04141975 | n03196217 | n03544143 |
| n04355338 | n03891332 | n04317175 | n04376876 | n03706229 | n02841315 | n04009552 | n04356056 | n03692522 | n04044716 |
| n02879718 | n02950826 | n02749479 | n04090263 | n04008634 | n03126707 | n03666591 | n02666196 | n02977058 | n04238763 |
| n03180011 | n03485407 | n03832673 | n06359193 | n03496892 | n04428191 | n04004767 | n04243546 | n04525305 | n04179913 |
| n03602883 | n04372370 | n03532672 | n02974003 | n03874293 | n03944341 | n03992509 | n03425413 | n02966193 | n04371774 |
| n04067472 | n04040759 | n04019541 | n03492542 | n04355933 | n03929660 | n02965783 | n04258138 | n04074963 | n03208938 |
| n02910353 | n03476684 | n03627232 | n03075370 | n03874599 | n03804744 | n04127249 | n04153751 | n03803284 | n04162706 |
| n04228054 | n02948072 | n03590841 | n04286575 | n04456115 | n03814639 | n03933933 | n04485082 | n03733131 | n03794056 |
| n04275548 | n01768244 | n01770081 | n01770393 | n01776313 | n01784675 | n01990800 | n01985128 | n01986214 | n02165456 |
| n02177972 | n02190166 | n02206856 | n02226429 | n02229544 | n02231487 | n02233338 | n02236044 | n02256656 | n02259212 |
| n02264363 | n02268443 | n02268853 | n02281787 | n01910747 | n01914609 | n01917289 | n01924916 | n01930112 | n01943899 |
| n01944390 | n01945685 | n01950731 | n01955084 | n02319095 | n02321529 | n03584829 | n03297495 | n03761084 | n03259280 |
| n04111531 | n04442312 | n04542943 | n04517823 | n03207941 | n04070727 | n04554684 | n03133878 | n03400231 | n04596742 |
| n02939185 | n03063689 | n04398044 | n04270147 | n02699494 | n04486054 | n03899768 | n04311004 | n04366367 | n04532670 |
| n02793495 | n03457902 | n03877845 | n03781244 | n03661043 | n02727426 | n02859443 | n03028079 | n03788195 | n04346328 |
| n03956157 | n04081281 | n03032252 | n03529860 | n03697007 | n03065424 | n03837869 | n04458633 | n02980441 | n04005630 |
| n03461385 | n02776631 | n02791270 | n02871525 | n02927161 | n03089624 | n04200800 | n04443257 | n04462240 | n03388043 |
| n03042490 | n04613696 | n03216828 | n02892201 | n03743016 | n02788148 | n02894605 | n03160309 | n04326547 | n03459775 |
| n04239074 | n04501370 | n03792972 | n04149813 | n03530642 | n03961711 | n03903868 | n02814860 | n07711569 | n07720875 |

***Subordinate-Level ImageNet Folder IDs***

| n02119789 | n02100735 | n02110185 | n02096294 | n02102040 | n02066245 | n02509815 | n02124075 | n02417914 | n02123394 |
| --- | --- | --- | --- | --- | --- | --- | --- | --- | --- |
| n02110063 | n02109047 | n02089867 | n02102177 | n02091134 | n02092002 | n02504458 | n02092339 | n02098105 | n02096437 |
| n02114712 | n02105641 | n02091635 | n02088466 | n02096051 | n02097130 | n02493509 | n02457408 | n02389026 | n02443484 |
| n02110341 | n02089078 | n02086910 | n02093256 | n02113978 | n02106382 | n02113712 | n02113186 | n02105162 | n02415577 |
| n02356798 | n02488702 | n02123159 | n02098413 | n02422699 | n02094433 | n02111277 | n02132136 | n02119022 | n02091467 |
| n02106550 | n02422106 | n02091831 | n02120505 | n02104365 | n02086079 | n02112706 | n02098286 | n02095889 | n02484975 |
| n02500267 | n02090721 | n02396427 | n02108000 | n02108915 | n02110806 | n02107683 | n02085936 | n02094114 | n02087046 |
| n02100583 | n02096177 | n02494079 | n02105056 | n02101556 | n02123597 | n02105505 | n02088094 | n02085782 | n02489166 |
| n02114548 | n02134084 | n02090622 | n02113624 | n02093859 | n02097298 | n02108551 | n02493793 | n02107142 | n02096585 |
| n02107574 | n02107908 | n02086240 | n02102973 | n02112018 | n02093647 | n02397096 | n02437312 | n02483708 | n02097047 |
| n02106030 | n02099601 | n02093991 | n02110627 | n02106166 | n02108089 | n02097658 | n02088364 | n02111129 | n02100236 |
| n02486261 | n02115913 | n02487347 | n02099849 | n02108422 | n02104029 | n02492035 | n02110958 | n02099429 | n02094258 |
| n02099267 | n02112350 | n02109961 | n02101388 | n02113799 | n02095570 | n02101006 | n02115641 | n02097209 | n02120079 |
| n02095314 | n02088238 | n02133161 | n02328150 | n02492660 | n02112137 | n02093428 | n02105855 | n02111500 | n02085620 |
| n02123045 | n02490219 | n02099712 | n02109525 | n02111889 | n02088632 | n02090379 | n02443114 | n02361337 | n02105412 |
| n02483362 | n02107312 | n02325366 | n02091032 | n02102318 | n02100877 | n02074367 | n02504013 | n02102480 | n02113023 |
| n02086646 | n02497673 | n02087394 | n02127052 | n02116738 | n02488291 | n02091244 | n02114367 | n02089973 | n02105251 |
| n02134418 | n02093754 | n02106662 | n01871265 | n03452741 | n02690373 | n04552348 | n03344393 | n04273569 | n03662601 |
| n04612504 | n04483307 | n03095699 | n03673027 | n03947888 | n04606251 | n03478589 | n03218198 | n02835271 | n03792782 |
| n03393912 | n03895866 | n03272562 | n04310018 | n02814533 | n03100240 | n04037443 | n04285008 | n03796401 | n03977966 |
| n04065272 | n03538406 | n03599486 | n03868242 | n02804414 | n03125729 | n03131574 | n03388549 | n03016953 | n03376595 |
| n07742313 | n07760859 | n11879895 | n04515003 | n03017168 | n04311174 | n02676566 | n03272010 | n11939491 | n12057211 |
| n09193705 | n09399592 | n09332890 | n01514668 | n01514859 | n01530575 | n01531178 | n01532829 | n01534433 | n01537544 |
| n01558993 | n01560419 | n01580077 | n01582220 | n01592084 | n01601694 | n01608432 | n01614925 | n01616318 | n01622779 |
| n01795545 | n01796340 | n01797886 | n01798484 | n01806143 | n01806567 | n01807496 | n01817953 | n01818515 | n01819313 |
| n01820546 | n01824575 | n01828970 | n01829413 | n01833805 | n01843065 | n01843383 | n01847000 | n01855032 | n01855672 |
| n01860187 | n02002556 | n02002724 | n02006656 | n02007558 | n02009912 | n02009229 | n02011460 | n02012849 | n02013706 |
| n02018207 | n02018795 | n02025239 | n02027492 | n02028035 | n02033041 | n02037110 | n02017213 | n02051845 | n02058221 |
| n01484850 | n01491361 | n01494475 | n01496331 | n01498041 | n02536864 | n01440764 | n01443537 | n02606052 | n02607072 |
| n02643566 | n02655020 | n02640242 | n02641379 | n01664065 | n01665541 | n01667114 | n01667778 | n01669191 | n01675722 |
| n01682714 | n01685808 | n01687978 | n01688243 | n01689811 | n01692333 | n01693334 | n01694178 | n01698640 | n01728572 |
| n01728920 | n01729322 | n01729977 | n01734418 | n01735189 | n01737021 | n01739381 | n01740131 | n01742172 | n01744401 |
| n01748264 | n01749939 | n01751748 | n01753488 | n01755581 | n01756291 | n01629819 | n01631663 | n01632458 | n01632777 |
| n01641577 | n01644373 | n01644900 | n02708093 | n04548280 | n04328186 | n03197337 | n03085013 | n04505470 | n01773157 |
| n01773549 | n01773797 | n01774384 | n01774750 | n01775062 | n01978287 | n01978455 | n01980166 | n01981276 | n01983481 |
| n01984695 | n02165105 | n02167151 | n02168699 | n02169497 | n02172182 | n02174001 | n02276258 | n02277742 | n02279972 |
| n02280649 | n02281406 | n03000134 | n03930313 | n04604644 | n07716906 | n07717410 | n07717556 | n07892512 |  |
